# Efficacy of elective nodal irradiation in skin squamous cell carcinoma of the face, ears, and scalp

**DOI:** 10.1186/s13014-015-0509-2

**Published:** 2015-09-21

**Authors:** Justin Wray, Robert J. Amdur, Christopher G. Morris, John Werning, William M. Mendenhall

**Affiliations:** Department of Radiation Oncology, University of Florida, 2000 SW Archer Road, PO Box 100385, Gainesville, FL USA; Department of Otolaryngology, University of Florida, Gainesville, FL USA

**Keywords:** Skin cancer, Head and neck, Radiotherapy, Elective nodal radiotherapy, Outcomes

## Abstract

**Background:**

In patients at high risk for regional node metastasis from squamous cell carcinoma (SCC) of the skin of the face, ear, or scalp, radiotherapy to the regional nodes is an alternative to parotid or neck surgery. Data on the efficacy of elective nodal radiotherapy in this setting are scarce such that there is no publication specifically addressing the subject. The purpose of our study is to fill this void in the skin cancer literature.

**Methods:**

This is a single-institution study of outcomes following elective nodal radiotherapy in 71 consecutively treated adults with SCC of the face, ears, or scalp. Primary site stage distribution per the American Joint Committee on Cancer, 7^th^ Edition, was as follows: T1, 15 %; T2, 34 %; T3, 1 %; and T4, 50 %. Other disease characteristics included the following: clinical perineural invasion, 13 %; pathological perineural invasion, 78 %; recurrent disease, 32 %; and positive or close margin, 67 %. The median radiation dose to the first- and second-echelon nodal area was 50 Gy. Acute and late toxicity were graded per the Common Terminology Criteria for Adverse Events, version 4.0. Regional control was assessed using the Kaplan-Meier product limit method.

**Results:**

Median followup was 4.5 years for all patients. The actuarial regional control rate at 5 years was 96 %. There were no (0 %) grade 3 or higher complications from elective nodal irradiation.

**Conclusions:**

Elective nodal irradiation in patients with high-risk SCC of the face, ears and scalp is safe and effective.

## Background

Major risk factors found at the primary site that predispose to nodal metastasis from squamous cell carcinoma (SCC) of the skin include the following: tumor diameter > 2 cm, tumor thickness > 5 mm, poor differentiation, tumor location within ear, age > 70 years, perineural invasion (PNI), history of recurrence, and immunosuppression [1, 2]. Elective neck surgery often including parotidectomy effectively prevents regional node recurrence, but the morbidity of surgery in this setting is frequently substantial [3, 4]. An alternative to elective neck surgery is elective nodal irradiation (ENI) since many of the cancers in which neck management is indicated also present with indications for primary or adjuvant radiation therapy (RT) to the primary site.

There is no study that focuses on ENI in SCC of the skin of the face, head, or neck. A few publications present subset analyses that address the ENI issue to some degree, but to our knowledge, none of these studies describe overall risk factors in the ENI group, the technical details of ENI, the actuarial risk of neck control following ENI, or toxicity related to ENI [5–7]. The purpose of this study is to fill this void in the skin cancer literature.

## Methods

Under the approval of the University of Florida Institutional Review Board, we reviewed the medical records of 71 patients with SCC of the skin of the face, ears, or scalp treated at our institution with RT to the primary site and regional lymphatics between 1985 and 2012. The year 1985 marks when our institution routinely began using axial computed tomography (CT) or magnetic resonance imaging (MRI) to stage head and neck cancer patients. This study is limited to patients who received elective nodal radiotherapy, meaning there was no clinical or radiographic evidence of nodal metastasis and no history of surgery to the regional lymphatics. Throughout the 28-year period of this study it was the policy in our department to add ENI in patients with recurrent tumors, poor differentiation, perineural invasion, tumor size greater than 2 cm, positive margin, or immunosuppression. This study is limited to situations wherein a major change in treatment volume was made specifically to electively irradiate the regional nodes. Not included in this study are cases wherein inclusion of nodal tissue was only accomplished by enlarging the primary field a small amount.

ENI was delivered to the first-echelon nodal region in all cases in this study. The distribution of first-echelon nodal areas among our cohort was as follows: parotid, 75 %; facial, 37 %; retroauricular, 28 %; occipital, 10 %; and cervical levels 1 and 2, 8 %.

Table 1 lists the relevant patient and tumor characteristics. Clinical PNI was defined as a cranial nerve deficit on physical examination and/or visible tumor in a major cranial nerve branch on CT or MRI scan.

**Table 1:** Patient characteristics (N = 71)

Characteristics	No. of patients (%)
Sex	
Male	48 (68)
Female	23 (32)
Race or ethnicity	
White	69 (97)
Black	1 (1.5)
Hispanic	1 (1.5)
Immunosuppressed	6 (8.5)
Adjuvant RT (post-op) without visible tumor	53 (75)
Visible tumor present at time of RT	18 (25)
Recurrent after curative-intent surgery	23 (32)
Clinical perineural invasion	9 (13)
Cancer touches midline	17 (24)
Radiological and Pathological Risk Factors	
>2 cm primary	36 (51)
> 5 mm thickness	24 (44)
>2 mm invasion	17 (31)
Subcutaneous fat invasion	10 (18)
Perineural invasion in pathology report	45 (76)
Lymph-vascular space invasion	8 (14)^a^
Bone invasion in pathology or imaging report	8 (12)^b^
Cartilage invasion in pathology or imaging report	6 (9)^b^
Margin status	
Positive margin	31 (56)^c^
Negative margin	18 (33)^c^
Close (< 5 mm) margin	6 (11)^c^
Differentiation	
Well differentiated	15 (21)
Moderately differentiated	14 (20)
Poorly differentiated	25 (35)
Undifferentiated	2 (3)
Not Reported	15 (21)
Nodes negative by CT or MR scan	60 (85)
Characteristics	Median value (range)
Age	69 years (33-95)
Days from previous treatment to recurrence	344 days (28-1078)

Notes: ^a^The percentage of patients was calculated with a total of 59 patients. ^b^The percentage of patients as calculated with a total of 68 patients. ^c^The percentage of patients was calculated with a total of 55 patients. Abbreviations: RT, radiation therapy; CT, computed tomography; MR, magnetic resonance

In addition to the information summarized in Table 1, distribution of primary site location was as follows: ear pinna (13 %), lateral cheek (11 %), temple (11 %), medial cheek (10 %), nose (10 %), external auditory canal (9 %), forehead (9 %), postauricular (7 %), posterior scalp (7 %), anterior scalp (5 %), upper lip vermillion (4 %), upper lip skin (3 %), and lower lip skin (1 %).

All patients in this study were clinically node-negative based on physical examination and 85 % by axial CT or MR scan. Primary site stage distribution per the American Joint Committee on Cancer, 7^th^ Edition [8], was as follows: T1, 15 %; T2, 34 %; T3 1 %; and T4 50 %. Patients were also staged according to a recently proposed, potentially more relevant staging system employing risk factors (poor differentiation, PNI, tumor diameter ≥2 cm, and invasion of subcutaneous fat) [6]. Stage distribution per this proposed system was as follows: T1 (0 risk factor), 6 %; T2A (1 risk factor), 34 %; T2B (2-3 risk factors), 43 %; and T3 (4 risk factors), 17 %.

Based on the data from Mendenhall [1], O’Hara [2], and Pahlajani [6], we estimate that the great majority of patients in our study had at least a 10 % chance of subclinical disease in the regional lymphatics. Table 2 summarizes the details of RT. In brief, the median radiation dose to the primary site was 65 Gy (range, 38-74 Gy), the median radiation dose to the first-echelon nodal area was 50 Gy (range, 38-74 Gy), and the median radiation dose to the other nodal areas was 50 Gy (range, 30-60 Gy). Three patients received concurrent chemotherapy for advanced disease with carboplatin and taxol (1 patient), carboplatin alone (1 patient) or cisplatin alone (1 patient).

**Table 2:** Radiation therapy (N = 71)

Treatment Characteristics	Median value (range)
Radiation therapy duration (days)	44 days (16 to 55)
Primary site dose	65 Gy (38 to 74)
1^st^ echelon node station dose	50 Gy (38 to 74)
Other node station dose	50 Gy (30 to 60)
Treatment Characteristic	No. of patients (%)
Primary site radiation therapy modality	
Orthovoltage (250-kV)	15 (21 %)
Electron	21 (30 %)
Cobalt-60	1 (1 %)
4- to 6-MV photon	20 (28 %)
Mixed photon-electron	14 (20 %)
First-echelon node radiation therapy modality	
Orthovoltage (250 kV)	11 (15 %)
Electron	24 (34 %)
Cobalt-60	0 (0 %)
4- to 6-MV photon	24 (34 %)
Mixed photon-electron	12 (17 %)
First-echelon node radiation therapy technique	
En-face Electron	24 (34 %)
En-face Mixed Electron-Photon	12 (17 %)
En-face Orthovoltage	11 (15 %)
6 MV Photon wedge pair	14 (20 %)
6 MV IMRT	7 (10 %)
Anterior 6MV Photon	3 (4 %)
Radiation therapy modality for other nodal stations	
Orthovoltage (250 kV)	0 (0)^a^
Electron	19 (32)^a^
Cobalt-60	4 (7)^a^
4- to 6-MV photon	34 (56)^a^
Mixed photon-electron	3 (5)^a^
Concurrent chemotherapy	3 (4)

Note: ^a^The percentage of patients as calculated with a total of 60 patients

### Statistical analyses

The efficacy endpoint in this study is regional control, which we define as freedom from tumor recurrence in a lymph node. All statistical analyses were performed using SAS and JMP software (SAS Institute, Cary, NC). Regional control was assessed using the Kaplan-Meier product limit method. The log-rank test statistic was used to detect any statistically significant differences between strata of selected explanatory variables.

The toxicity endpoint in this study is an effect that could be related to elective nodal RT. We graded acute and late toxicities with the most recent version of the National Cancer Institute Common Terminology Criteria for Adverse Events, version 4.0 (CTCAE v4.0) [7]. In this retrospective analysis we were not able to accurately report minor (grade 1 or 2) toxicities. For this reason our report is limited to grade 3 to 5 toxicities.

## Results

### Follow-up

The median follow-up after the last day of RT was 4.5 years for all patients (range, 0.8-22.5 years), and 6.0 years for living patients (range, 1.9-22.5 years).

### Regional control after elective nodal irradiation

The rate of isolated nodal recurrence in an area treated with ENI was 2 of 71 (2.8 %). Table 3 summarizes the details of these two cases.

**Table 3:** Characteristics of the 2 patients with isolated nodal recurrence related to elective nodal irradiation

Characteristics	Patient 1	Patient 2
Age (years)	64	75
Subsite	Pinna	Temple
First echelon treated	Parotid	Parotid
Extended elective nodal irradiation	Facial; cervical 1b and 2	Cervical 2
Sex	Male	Male
Race	White	White
American Joint Committee on Cancer, 7th ed., stage	T4	T1
Proposed 2013 Journal of American Medical Association stage^7^	T2A	T2A
Axial imaging performed	Yes	Yes
Immunosuppressed	Yes	No
Cancer touches midline	No	No
Clinical perineural invasion	No	No
Pathological perineural invasion	Yes	No
>2 cm primary	No	No
>5 mm thickness	No	No
>2 mm invasion	No	No
Invaded subcutaneous fat	No	No
Lymphovascular space invasion	Yes	No
Bone invasion on pathology or imaging	No	No
Cartilage invasion on pathology or imaging	No	No
Recurrent after curative-intent surgery	Yes	Yes
Days from first surgery to radiotherapy start	244	323
Positive or close margin	Yes	No
Histology	Moderate	Poor
Adjuvant (postoperative) without visible tumor	Yes	Yes
Primary (Gy)	60 (once daily)	74 (twice daily)
1st Echelon (Gy)	46 (once daily)	74 (twice daily)
Rest (Gy)	50 (once daily)	46 (once daily)

In addition to 2 isolated nodal recurrences in areas of ENI, 2 patients experienced nodal recurrence that we do not attribute to ENI. One patient developed a nodal recurrence in a contralateral level 1 node outside of the RT target volume and would not have been removed through an elective neck dissection; therefore, we do not consider this event a failure of ENI compared to the surgical alternative. An additional patient simultaneously recurred in the primary site and regional node, which suggests that the nodal recurrence could be a secondary event related to metastasis from the primary site recurrence rather than failure of the ENI to sterilize subclinical disease.

Fifteen patients recurred at the primary site (of these, 1 had a simultaneous nodal recurrence in the first-echelon nodes and 14 never developed a nodal recurrence throughout the follow-up period). The most reliable measure of efficacy of ENI is the regional recurrence rate in patients who received ENI but remained continuously free of recurrence at the primary site and the area which received ENI: In our study, 2 of 56 patients recurred in this area (3.6 %).

**Fig. 1:**
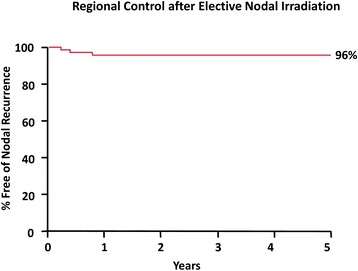
Kaplan-Meier curve of neck control with (n = 78) and without (n = 56) local control

Figure 1 is an actuarial plot of regional control following ENI. After rounding off to the nearest whole number, the actuarial 5-year rate of continuous freedrom from a nodal recurrence (not including surgical salvage) was 96 % for both the overall group of 71 patients and for the 56 patients without a primary site recurrence.

### Time to nodal recurrence after Elective Nodal Irradiation

All 4 nodal recurrences presented within 1 year of completing RT.

### Regional control including surgical salvage of nodal recurrence

In 3 of the 4 patients with a nodal recurrence, salvage neck dissection was performed with curative intent (and the patient who simultaneously recurred at the primary site was also treated with resection of the primary site). Salvage surgery was successful in 2 of the 3 patients who underwent salvage attempt based on no evidence of cancer at last follow-up (at least 1 year after salvage surgery). In the remaining patient, salvage surgery was not attempted because of the extent of adenopathy and underlying medical problems.

### Toxicity of Elective Nodal Irradiation

There were no (0 %) grade 3 or higher toxicity events that could be related to ENI.

## Discussion

The primary value of this series is that it is the first study to focus on the efficacy of ENI in a general population of patients with SCC of the skin of the head and neck, for whom this issue is most pertinent. We know of only 3 other studies with data on this subject and none include details about overall risk factors, ENI target areas, ENI dose, or location of recurrence relative to ENI [5, 9, 10].

The 1987 study by Mendenhall et al from our department reported outcomes of patients treated with radiotherapy for gross disease at the primary site [9]. Almost half the patients had basal cell carcinoma and most were not staged with cross-sectional imaging. The only data relevant to our discussion is that, with ENI, 8 of 10 patients with recurrent SCC remained continuously free of a nodal recurrence.

The 2005 study by Moore and colleagues reports freedom from recurrence in 4 of 5 patients treated with ENI, but it is unclear if the sole recurrence was local, regional, or both [5]. In patients who present with parotid gland metastases, cervical ENI has been previously shown to decrease neck failures from 50 % in observed patients to 0 % in those treated with ENI [11]. The more recent series from our department authored by Balamucki and colleagues focuses on outcomes of patients with clinical or incidental PNI from basal or squamous cell carcinoma of the skin [10]. With ENI, the actuarial rate of regional control at 5 years was 96 % in patients with clinical PNI and 100 % in patients with incidental PNI.

## Conclusions

The experience reported in this paper supports the conclusion that, in patients with SCC of the skin of the face, pinna, external auditory canal, or scalp, the rate of nodal recurrence and toxicity is very low in areas that receive at least 50 Gy of ENI. The implication of this conclusion is that, in patients similar to the study population, clinicians should consider ENI when the risk of subclinical disease in the regional lymphatics is considered to be high. This recommendation is supported by the 2015 guidelines from the National Comprehensive Cancer Center Network which include ENI (50 Gy at 2 Gy per fraction) in patients with SCC of the skin who are “at risk for subclinical disease” and have not undergone elective neck dissection [12].

The nodal areas we treated in this study are listed in Table 4. As this study was limited to patients who received ENI, the data does not inform the question of indications for elective treatment of the regional nodes, how an approach using ENI compares to elective neck dissection, or observation with treatment reserved for salvage of nodal recurrence.

**Table 4:** First echelon nodal volumes treated and the techniques used.

Nodal Area (%)	Most Common First Echelon Nodal Area Treated (%)	Most Common Radiation Technique Used (%)	2nd Most Common Radiation Technique Used (%)	3rd Most Common Radiation Technique Used (%)
Ear Pinna (13)	Parotid (100)	Photons (44)	Electrons (22)	Mixed (22)
Lateral Cheek (11)	Parotid (100)	Photons (38)	Mixed (38)	Electrons (13)
Medial Cheek (10)	Parotid (100)	Photons (29)	Electrons (29)	Mixed (29)
Temple (11)	Parotid (100)	Electrons (75)	Mixed (13)	Photons (13)
Nose (10)	Facial (71)	Orthovoltage (60)	Photons (40)	NA
External Auditory Canal (9)	Parotid (100)	Photons (67)	Electrons (17)	Mixed (17)
Forehead (9)	Parotid (83)	Electrons (100)		
Post-auricular (7)	Retro-auricular (100)	Photons (40)	Orthovoltage (40)	Mixed (20)
Posterior Scalp (7)	Occipital (80)	Photons (50)	Mixed (50)	NA
	Post-auricular (80)	Mixed (50)	Electrons (25)	Photons (25)
Anterior Scalp (5)	Parotid (75)	Electrons (100)	NA	NA
Upper lip vermillion (4)	Bilateral Cervical (66)	Photons (100)	NA	NA
Upper lip skin (3)	Facial (100)	Photons (50)	Orthovoltage (50)	
Lower lip skin (1)	Ipsilateral Cervical (100)	Photons (100)	NA	NA

NA, not available

Mixed: includes the use of both electrons, orthovoltage and photons to achieve coverage of the indicated nodal area
